# Supplementary material for: Content-rich biological network constructed by mining PubMed abstracts
Source: BMC Bioinformatics. 2004 Oct 8;5:147. doi: 10.1186/1471-2105-5-147 (PMC528731; doi:10.1186/1471-2105-5-147)
Supplement: Additional File 2 — The original results of the above study (non-essential files are deleted to keep the file size under the limit set by BMC bioinformatics). [file 1471-2105-5-147-S2.bz2 › chilibotAdditionalFile2/dip20/313ID10606357E1370/html/S100A8_S100A9.html]

 


 **S100A8** and **S100A9** 
  
Found 177 abstracts in PubMed, retrieved 20.  
 

 What does Google say? 
 PDF only 
| .edu only 

---

**Interactive relationship** (e.g. stimulation, inhibition, etc)

**Stimulatory relationship**- An abundant 32kDa protein capable of stimulating IL 8 production was isolated from the secretion and identified as neutrophil cytoplasmic protein MRP 14  [ **S100A9** ] , which is the heavy polypeptide chain in the MRP 8  [ **S100A8** ]  14 heterodimer.  Ref: 12748056 Am J Respir Cell Mol Biol< MedlineTA>Am J Respir Cell Mol Biol,
**Neutral relationship**- MRP 8  [ **S100A8** ]  mRNA but not protein is present in the myeloid cells of these mice, suggesting that the stability of MRP 8  [ **S100A8** ]  protein is dependent on MRP 14  [ **S100A9** ]  expression.  Ref: 12640137 Mol Cell Biol, 2003
- Moreover, MRP8  [ **S100A8** ]  MRP14  [ **S100A9** ]  initiated oxidase activation on its own, through a calcium dependent specific interaction with cytochrome b558 as shown by Atomic Force Microscopy and a structure function relationship investigation.  Ref: 12719414 J Biol Chem< MedlineTA>J Biol Chem, 2003
- There was also an upregulation of  **S100A8**  A9 or A12 protein, as detected by the antibody MAC387  [ **S100A9** ] , in normal keratinocytes adjacent to both atypical keratinocytes and melanocytes in PAK and LM, respectively.  Ref: 12652189 Am J Dermatopathol, 2003

**Non-interactive relationship** (e.g. studied together, co-existance, homology, etc.)

- It was also possible to detect fibronectin networks and MRP8  [ **S100A8** ] , MRP14  [ **S100A9** ] , and defensin positive granulocytes and macrophages in particular wounds up to 30 days old.  Ref: 12590055 Forensic Sci Int, 2003
- OBJECTIVES To determine whether myeloid related proteins MRP8  [ **S100A8** ]  MRP14  [ **S100A9** ] , a complex of two S100 proteins related to neutrophil and monocyte activation, might be used as a marker for disease activity, and as an early indicator of relapse in juvenile idiopathic arthritis.  Ref: 12594109 Ann Rheum Dis, 2003
- A compensatory increase in other proteins was not detected in cells lacking MRP 8  [ **S100A8** ]  and MRP 14  [ **S100A9** ] .  Ref: 12640137 Mol Cell Biol, 2003
- ... S100A7,  **S100A8** , and  **S100A9**  are markedly overexpressed.  Ref: 12704215 J Histochem Cytochem, 2003
- Calprotectin  **S100A8**   **S100A9** , an Inflammatory Protein Complex from Neutrophils with a Broad Apoptosis Inducing Activity.  Ref: 12808281 Biol Pharm Bull, 2003
- These studies 1 show that  **S100A8** ,  **S100A9** , and  **S100A8**  A9 are potent stimulators of neutrophils and 2 strongly suggest that these proteins are involved in neutrophil migration to inflammatory sites.  Ref: 12626582 J Immunol, 2003
- **S100A9**   **S100A8**  Myeloid representatives of the S100 protein family as prominent players in innate immunity.  Ref: 12645005 Microsc Res Tech, 2003
- In a semi recombinant cell free assay, recombinant MRP8  [ **S100A8** ]  MRP14  [ **S100A9** ]  increased the affinity of p67 phox for cytochrome b558 synergistically with p47 phox.  Ref: 12719414 J Biol Chem< MedlineTA>J Biol Chem, 2003
- **S100A8** , the heterodimerization partner of  **S100A9**  was not detectable in peripheral blood cells, suggesting that even a deficiency in both  **S100A8**  and  **S100A9**  proteins was compatible with viable and mature neutrophils.  Ref: 12529407 Mol Cell Biol< MedlineTA>Mol Cell Biol, 2003
- During a transient relapse there was an increase in MRP8  [ **S100A8** ]  MRP14  [ **S100A9** ] .  Ref: 12594109 Ann Rheum Dis, 2003
- Expression of MRP8  [ **S100A8** ] , MRP14  [ **S100A9** ] , and MRP8  [ **S100A8** ]  MRP14  [ **S100A9** ]  in synovium was determined by immunohistochemistry.  Ref: 12794836 Arthritis Rheum, 2003
- CONCLUSIONS MRP8  [ **S100A8** ]  MRP14  [ **S100A9** ]  serum concentration can be used as a marker for disease activity in patients who receive an ASCT for refractory JIA.  Ref: 12594109 Ann Rheum Dis, 2003
- A novel inflammatory syndrome defined by extraordinarily high expression of  **S100A8**  and  **S100A9**  confirmed recent observations in vitro demonstrating a role of these proteins during recruitment of leukocytes.  Ref: 12697438 Trends Immunol, 2003
- Changing the conformation state of cytochrome b558 initiates NADPH oxidase activation MRP8  [ **S100A8** ]  MRP14  [ **S100A9** ]  regulation.  Ref: 12719414 J Biol Chem< MedlineTA>J Biol Chem, 2003
- **S100A8**  and  **S100A9**  are absent or are expressed at minimal levels in normal epidermis.  Ref: 12704215 J Histochem Cytochem, 2003
- The presence of an active phenotype of macrophages was studied by detection of myeloid related proteins 8 and 14 MRP8  [ **S100A8** ]  and MRP14  [ **S100A9** ] , respectively.  Ref: 12528127 Arthritis Rheum, 2003
- However, detection of MRP8  [ **S100A8** ] , MRP14  [ **S100A9** ]  and defensin provides no advantage over the routine histological detection of granulocytes and macrophages in wounds under 1 2 days old.  Ref: 12590055 Forensic Sci Int, 2003
- These studies also indicate, as reported previously, that S100A7,  **S100A8** , and  **S100A9**  expression is markedly altered in psoriasis, suggesting a role for these proteins in disease pathogenesis.  Ref: 12704215 J Histochem Cytochem, 2003
- RESULTS After ASCT, MRP8  [ **S100A8** ]  MRP14  [ **S100A9** ]  serum concentrations in JIA showed a positive correlation with the Child Health Assessment Questionnaire CHAQ.  Ref: 12594109 Ann Rheum Dis, 2003
- In addition, elevated levels of  **S100A9**  and  **S100A8**  in body fluids of inflamed tissues strengthen the view that these molecules are important players in fighting inflammation.  Ref: 12645005 Microsc Res Tech, 2003
- **S100A8**  and  **S100A9**  are strongly expressed in the basal and spinous layers in psoriasis involved tissue.  Ref: 12704215 J Histochem Cytochem, 2003
- MRP8  [ **S100A8** ]  MRP14  [ **S100A9** ]  purified from neutrophils, enhanced oxidase turnover of B cells in vitro, suggesting that MRP8  [ **S100A8** ]  MRP14  [ **S100A9** ]  is involved in the activation process.  Ref: 12719414 J Biol Chem< MedlineTA>J Biol Chem, 2003
- **S100A8**  and  **S100A9**  did not form these larger complexes.  Ref: 12704215 J Histochem Cytochem, 2003
- Concentrations of MRP8  [ **S100A8** ]  MRP14  [ **S100A9** ]  in serum and SF were measured by enzyme linked immunosorbent assay.  Ref: 12794836 Arthritis Rheum, 2003
- The time dependent expression of fibronectin, MRP8  [ **S100A8** ] , MRP14  [ **S100A9** ]  and defensin in surgically treated human skin wounds.  Ref: 12590055 Forensic Sci Int, 2003
- **S100A8**  and  **S100A9**  calcium binding proteins localization within normal and cyclosporin A induced overgrowth gingiva. **S100A8**  and  **S100A9** , also called myeloid related protein 8 and 14, are calcium binding proteins highly expressed in neutrophils, in which they play a key role in the inflammatory progression.  Ref: 12489193 Connect Tissue Res, 2002
- No differences between the expression of MRP8  [ **S100A8** ]  and MRP14  [ **S100A9** ]  could be demonstrated in the wounds, the majority of which were only several hours old.  Ref: 12590055 Forensic Sci Int, 2003
- Mean MRP8  [ **S100A8** ]  MRP14  [ **S100A9** ]  serum concentrations dropped markedly in the first three months after ASCT p=0.0039 and clinical parameters of disease activity such as CHAQ markedly improved p=0.0039.  Ref: 12594109 Ann Rheum Dis, 2003
- Finally, injection of  **S100A8** ,  **S100A9** , or  **S100A8**  A9 into a murine air pouch model led to rapid, transient accumulation of neutrophils confirming their activities in vivo.  Ref: 12626582 J Immunol, 2003
- Analyses by means of reverse transcription polymerase chain reaction showed enhanced expression of several genes including calgranulin A  [ **S100A8** ] , calgranulin B  [ **S100A9** ] , psoriasin, thymosin beta 10, cathepsin C, cathepsin D, and cathepsin H in cholesteatoma, supporting the findings from the gene array.  Ref: 12792315 Laryngoscope, 2003
- RESULTS MRP8  [ **S100A8** ] , MRP14  [ **S100A9** ] , and MRP8  [ **S100A8** ]  MRP14  [ **S100A9** ]  levels were increased in serum, SF, and synovium from PsA, RA, and SpA patients.  Ref: 12794836 Arthritis Rheum, 2003
- In all 3 groups, paired samples of serum and SF showed significantly higher MRP8  [ **S100A8** ]  MRP14  [ **S100A9** ]  levels in SF mean SD 1 10 1 99 ng ml median 1 00 than in serum 908 679 ng ml median 695 P = 0.0001.  Ref: 12794836 Arthritis Rheum, 2003
- Together with  **S100A8**  and  **S100A9** , it belongs to the calgranulin subfamily, i.e. it is mainly expressed in granulocytes, although there is an increasing body of evidence of expression in keratinocytes and psoriatic lesions.  Ref: 12777802 Acta Crystallogr D Biol Crystallogr, 2003
- MRP8  [ **S100A8** ]  and MRP14  [ **S100A9** ]  are absent from Epstein Barr virus immortalized B lymphocytes and coincidentally, these cells display weak oxidase activity compared with neutrophils.  Ref: 12719414 J Biol Chem< MedlineTA>J Biol Chem, 2003
- Granulocytic infiltrates limited to the perivasal space could be detected 20 30 min after infliction of the wound expressing MRP8  [ **S100A8** ] , MRP14  [ **S100A9** ]  and defensin.  Ref: 12590055 Forensic Sci Int, 2003
- The occurrence of MAS in three patients in this study was not preceded by significant changes in MRP8  [ **S100A8** ]  MRP14  [ **S100A9** ]  concentration.  Ref: 12594109 Ann Rheum Dis, 2003
- MRP8  [ **S100A8** ]  and MRP14  [ **S100A9** ]  were co isolated from neutrophils on an anti p47 phox matrix with oxidase cytosolic factors and identified by mass spectrometry.  Ref: 12719414 J Biol Chem< MedlineTA>J Biol Chem, 2003
- Fibronectin, MRP8  [ **S100A8** ] , MRP14  [ **S100A9** ]  and defensin were detected immunohistochemically in 46 surgically treated, fresh hours old human wounds and in wounds of 13 individuals who died immediately from fatal trauma airplane crashes or train rollovers.  Ref: 12590055 Forensic Sci Int, 2003
- MRP8  [ **S100A8** ]  MRP14  [ **S100A9** ]  levels in serum correlated with systemic parameters of disease activity erythrocyte sedimentation rate ESR r = 0.55, P = 0.005.  Ref: 12794836 Arthritis Rheum, 2003
- Myeloid related protein 14 MRP 14  [ **S100A9** ]  and its heterodimeric partner, MRP 8  [ **S100A8** ] , are cytosolic calcium binding proteins, highly expressed in neutrophils and monocytes.  Ref: 12640137 Mol Cell Biol, 2003
- In this study, we report that  **S100A8** ,  **S100A9** , and  **S100A8**  A9 caused neutrophil chemotaxis at concentrations of 10 12 10 9 M.  Ref: 12626582 J Immunol, 2003
- Proinflammatory Activities of S100 Proteins  **S100A8** ,  **S100A9** , and  **S100A8**  A9 Induce Neutrophil Chemotaxis and Adhesion.  Ref: 12626582 J Immunol, 2003
- Our present studies compare the level of expression, and cellular localization of S100A7,  **S100A8** ,  **S100A9** , S100A10, and S100A11 in normal and psoriatic epidermis.  Ref: 12704215 J Histochem Cytochem, 2003
- **S100A8**  and  **S100A9**  are small calcium binding proteins that are highly expressed in neutrophil and monocyte cytosol and are found at high levels in the extracellular milieu during inflammatory conditions.  Ref: 12626582 J Immunol, 2003
- Myeloid related proteins MRP8  [ **S100A8** ]  and MRP14  [ **S100A9** ]  of the S100 family are EF hand calcium binding proteins.  Ref: 12719414 J Biol Chem< MedlineTA>J Biol Chem, 2003
- Two S100 proteins, namely  **S100A9**  MRP14  [ **S100A9** ]  and  **S100A8**  MRP8  [ **S100A8** ] , constitute roughly 40% of the cytosolic protein in these cells, implying by their pure abundance an important role in the effector functions of neutrophils.  Ref: 12645005 Microsc Res Tech, 2003
- In immediate fatal trauma, it was not possible to detect fibronectin outside of bleeding areas nor could inflammatory cells be visualized in the interstitium using MRP8  [ **S100A8** ] , MRP14  [ **S100A9** ] , or defensin antibodies.  Ref: 12590055 Forensic Sci Int, 2003
- One reason might be the conformational variability the  **S100A9**  and  **S100A8**  molecules can adopt.  Ref: 12645005 Microsc Res Tech, 2003
- The aim of this review is to give an update on the current developments concerning the  **S100A9**   **S100A8**  molecule in biology and medicine.  Ref: 12645005 Microsc Res Tech, 2003
- This was confirmed ex vivo by co transfection of EBV transformed B lymphocytes with genes encoding MRP8  [ **S100A8** ]  and MRP14  [ **S100A9** ] .  Ref: 12719414 J Biol Chem< MedlineTA>J Biol Chem, 2003
- In contrast, MRP8  [ **S100A8** ] , MRP14  [ **S100A9** ] , and MRP8  [ **S100A8** ]  MRP14  [ **S100A9** ]  expression in the SLL of PsA patients is increased, particularly in perivascular regions, compared with that in RA and SpA patients, suggesting a central role of MRP proteins in transendothelial migration of leukocytes in PsA.  Ref: 12794836 Arthritis Rheum, 2003
- Moreover, MRP8  [ **S100A8** ]  MRP14  [ **S100A9** ]  dimer behaves as a positive mediator of phagocyte NADPH oxidase regulation.  Ref: 12719414 J Biol Chem< MedlineTA>J Biol Chem, 2003
- Protein complexes formed by  **S100A8**  and  **S100A9**  represent the only AA binding capacity in the human neutrophilic cytosol and are involved in the intracellular arachidonic acid metabolism.  Ref: 12553726 Biol Chem, 2002
- In addition, it was verified by immunohistochemical analysis that the expressions of Calgranulin A  [ **S100A8** ] , Calgranulin B  [ **S100A9** ] , and Cathepsin D were mainly located in cholesteatoma epithelium.  Ref: 12792315 Laryngoscope, 2003
- Together with two other calgranulins,  **S100A8**  and  **S100A9** , it is mostly expressed in human granulocytes, although there is increasing evidence of expression in keratinocytes and psoriatic lesions.  Ref: 12645006 Microsc Res Tech, 2003
- OBJECTIVE To analyze S 100 protein expression, in the form of myeloid related protein 8 MRP8  [ **S100A8** ] , MRP14  [ **S100A9** ] , and the heterodimer MRP8  [ **S100A8** ]  MRP14  [ **S100A9** ] , in psoriatic arthritis PsA patients compared with rheumatoid arthritis RA and spondylarthropathy SpA patients, and to determine the effect of methotrexate MTX on the MRP antigen expression in PsA patients.  Ref: 12794836 Arthritis Rheum, 2003
- MRP8  [ **S100A8** ]  MRP14  [ **S100A9** ]  serum concentrations were determined by a sandwich enzyme linked immunosorbent assay ELISA as described.  Ref: 12594109 Ann Rheum Dis, 2003
- However, neutrophil activation by  **S100A8** ,  **S100A9** , and  **S100A8**  A9 did not induce actin polymerization.  Ref: 12626582 J Immunol, 2003
- **S100A8** ,  **S100A9** , and  **S100A8**  A9 stimulated shedding of L selectin, up regulated and activated Mac 1, and induced neutrophil adhesion to fibrinogen in vitro.  Ref: 12626582 J Immunol, 2003
- These included genes involved in cell proliferation and differentiation eg, calgranulin A  [ **S100A8** ] , calgranulin B  [ **S100A9** ] , psoriasin, thymosin beta 10 and cell invasion eg, cathepsin C, cathepsin D, cathepsin H.  Ref: 12792315 Laryngoscope, 2003
- The data indicate that MRP 14  [ **S100A9** ]  and MRP 8  [ **S100A8** ]  are dispensable for many myeloid cell functions.  Ref: 12640137 Mol Cell Biol, 2003
